# Experiences of gender-based violence among female sex workers, men who have sex with men, and transgender women in Latin America and the Caribbean: a qualitative study to inform HIV programming

**DOI:** 10.1186/s12914-019-0187-5

**Published:** 2019-03-05

**Authors:** Emily Evens, Michele Lanham, Karin Santi, Juana Cooke, Kathleen Ridgeway, Giuliana Morales, Caleb Parker, Claire Brennan, Marjan de Bruin, Pavel Chladni Desrosiers, Xenia Diaz, Marta Drago, Roger McLean, Modesto Mendizabal, Dirk Davis, Rebecca B. Hershow, Robyn Dayton

**Affiliations:** 1 FHI 360, 359 Blackwell Street, Durham, NC 27707 USA; 2 United Nations Development Programme, Panama City, Panama; 3 University of the West Indies, Mona Campus, Kingston, Jamaica; 4 LINKAGES Project, FHI 360, Port-au-Prince, Haiti; 5 United Nations Development Programme, San Salvador, El Salvador; 6 Centre for Health Economics, The University of the West Indies St. Augustine Campus, St Augustine, Trinidad and Tobago; 7 Asociación Diké de Hombres y Mujeres Transgénero y LGBTI+, San Salvador, El Salvador; 8 Gillings School of Global Public Health, University of North Carolina, Chapel Hill, USA

**Keywords:** Key populations, Sex workers, Men who have sex with men, Transgender women, Qualitative, HIV, Gender-based violence, Gender, Human rights, Latin American and the Caribbean

## Abstract

**Background:**

Female sex workers, MSM, and transgender women—collectively referred to as key populations (KPs)—are disproportionately affected by gender-based violence (GBV) and HIV, yet little is known about the violence they face, its gender-based origins, and responses to GBV. The purpose of this study was to understand the nature and consequences of GBV experienced, to inform HIV policies and programming and to help protect KPs’ human rights.

**Methods:**

Using a participatory approach, FSWs, MSM, and transgender women in Barbados, El Salvador, Trinidad and Tobago, and Haiti conducted 278 structured interviews with peers to understand their experiences of and responses to GBV. Responses to open-ended questions were coded in NVivo and analyzed using an applied thematic analysis.

**Results:**

Nearly all participants experienced some form of GBV. Emotional and economic GBV were the most commonly reported but approximately three-quarters of participants reported sexual and physical GBV and other human rights violations. The most common settings for GBV were at home, locations where sex work took place such as brothels, bars and on the street; public spaces such as parks, streets and public transport, health care centers, police stations and—for transgender women and MSM—religious settings and schools. The most common perpetrators of violence included: family, friends, peers and neighbors, strangers, intimate partners, sex work clients and other sex workers, health care workers, police, religious leaders and teachers. Consequences included emotional, physical, and sexual trauma; lack of access to legal, health, and other social services; and loss of income, employment, housing, and educational opportunities. Though many participants disclosed experiences of GBV to friends, colleagues and family, they rarely sought services following violence. Furthermore, less than a quarter of participants believed that GBV put them at risk of HIV.

**Conclusions:**

Our study found that across the four study countries, FSWs, MSM, and transgender women experienced GBV from state and non-state actors throughout their lives, and much of this violence was directly connected to rigid and harmful gender norms. Through coordinated interventions that address both HIV and GBV, this region has the opportunity to reduce the national burden of HIV while also promoting key populations’ human rights.

## Background

The HIV epidemic in Latin American and the Caribbean—as in many other regions—is mainly concentrated among female sex workers (FSWs), gay men and other men who have sex with men (MSM), and transgender women—collectively referred to as key populations (KPs) affected by HIV [1]. While the HIV prevalence among the adult population in Latin America is estimated at around 0.4% and in the Caribbean at 1.1%, the prevalence is much higher among KPs [2]. In El Salvador for example, where the HIV prevalence among the adult population is estimated at around 0.8%, the prevalence is 3.1% among sex workers, 10.5% among MSM, and 19% among transgender women [3]. Similarly, in Jamaica, the HIV prevalence rate in the general adult population is estimated at 1.6%, while prevalence among FSWs is estimated at 4.1%, prevalence among MSM is estimated at 31.4%, and estimates of prevalence among transgender women range between 25.2 and 52.9% [4–7].

Key populations in Latin America and the Caribbean also experience high levels of gender-based violence (GBV), defined as any violence directed at an individual based on their biological sex, gender identity (e.g., transgender), or behaviors that are inconsistent with social expectations of “being” a man or woman [8]. Gender-based violence includes emotional, sexual, physical, or economic abuse or discrimination by state and non-state actors, and violates the fundamental human right to live free from violence [9, 10]. Although data are lacking in many countries, global and regional studies show that GBV against FSW, MSM, and transgender women is prevalent, frequent, and often severe [11–18]. For example, 31% of MSM surveyed in the Caribbean and 34% of those surveyed in Latin America in 2014 had been physically assaulted due to their sexual orientation [19], and data from 2014 found that 28% of MSM and transgender women (data not disaggregated) experienced psychological abuse in San Salvador in the previous 12 months [17]. Individuals who are members of multiple KP groups, such as transgender sex workers, are disproportionately affected by GBV [20].

An increasing body of global research links experiences of GBV to increased HIV risk through intermediate risk factors including: multiple sex partners, coerced sex, substance use, unprotected sex, poor access to health services, lack of access to justice, and negative mental health and emotional repercussions such as suicidal behaviors, depression, and social isolation [12, 17, 21–26]. Moreover, GBV affects KPs’ uptake of and adherence to antiretroviral treatment [27–30], and discrimination and abuse from healthcare providers has been found to be a barrier to accessing HIV-related services among sex workers, MSM and transgender women [23, 31–33]. In resource-constrained settings, such as those that presently exist in Latin America and the Caribbean, the effects of GBV could reverse the gains achieved against HIV and derail the response to the epidemic in the region.

While we know the experience of GBV among FSWs, MSM, and transgender women is common, relatively little is known about the nature of GBV across KPs’ lives, whether and to whom KPs disclose experiences of GBV, which services KP members access when GBV occurs, and KP members’ perspectives regarding how HIV programs could increase their responsiveness to KP victims of GBV, especially in the Latin American and Caribbean region. Moreover, previous research has often treated MSM and transgender women as one group, failing to explore distinct experiences and risks unique to each [17]. Additionally, research on violence among KPs has generally focused on physical and sexual violence while emotional and economic violence and human rights violations have not been extensively explored. [34]. Finally, most previous research explores recent violence perpetrated by specific actors such as police or sex work clients and does not take a life course perspective or explore the gender-based origins of violence [34]. Achieving epidemic control and reaching the 90–90-90 goals requires that the HIV epidemic be better addressed among key populations [35]. Understanding where and when members of KPs face GBV, what they do after GBV occurs, and how HIV programs can better integrate responses to GBV into their programming are central to controlling the HIV epidemic, developing HIV policies and programs that are more effective and responsive to the needs of KPs, and realizing KPs’ human rights.

This research was intended to inform programs and policies to more effectively prevent and respond to GBV against members of key populations in Latin American and the Caribbean collectively. The purpose of this study was to generate high-quality evidence on the nature of GBV experienced by FSWs, MSM, and transgender women, to describe the consequences of and responses to GBV from the perspective of KP members, and to inform HIV service delivery policies and programming in Latin America and the Caribbean by making it more responsive to the needs of KP victims of GBV.

## Methods

Adapting the methodology used by *The Right(s) Evidence: Sex work, violence and HIV in Asia* [36], The LINKAGES project, United Nations Development Programme (UNDP), and The University of the West Indies (UWI) worked with local organizations providing services to KPs to collect data on experiences of GBV among FSW, MSM, and transgender women in San Salvador, El Salvador; Port of Spain, Trinidad and Tobago; Bridgetown, Barbados; and Ouanaminthe, Jacmel, and Port Au Prince, Haiti in 2016. The study team used two criteria to identify study sites: 1) the presence of local KP networks interested in this work, and 2) interest in addressing GBV among KP groups from the government, civil society, United Nations (UN), USAID Washington, and USAID country missions. In Haiti, three cities were included because they represented potentially different risk environments for KPs and additional funding was available.

A qualitative, participatory approach was used to identify FSWs, MSM, and transgender women study participants and explore GBV, a sensitive subject. Participatory research aims to involve those traditionally seen as subjects in generating, validating and using knowledge and creates a partnership between social groups and the scientific community to yield information that is more legitimate and useful for social change. Key populations were directly involved with the intention of increasing the quality and credibility of data, empowering KPs to conduct research, and ensuring the study was responsive to KPs’ interests and needs. Members of KP groups were actively engaged throughout the research process, including study design, developing interview guides, selecting sites, recruiting participants, conducting interviews, and interpreting study results. For example, KP members decided which contexts would be covered in the interviews: FSW representatives wanted to collect data on workplace violence but did not wish to ask about violence experienced before the age 18 or from an intimate partner, while transgender women and MSM representatives felt that these contexts were important to include. It should be noted that some FSWs did spontaneously disclose GBV before the age of 18 and GBV from a partner in response to open-ended questions.

Additionally, to facilitate collaboration with regional and national actors and ensure they could function as key partners for translating study results into action, the study team also formed regional and national advisory groups—including civil society organizations, UN agencies, USAID, UWI, government representatives, and the study team—to discuss the content and procedures for data collection. Sample sizes were derived according to norms for qualitative data collection to ensure data saturation, the point at which no new information is added by additional participants. [37]. Additionally, during analysis, the study team reviewed data and held discussions to ensure saturation had occurred. As this was qualitative research, results were not designed to be statistically representative of the study populations in each country. Fifteen FSW and transgender women were interviewed in each study site (El Salvador, Trinidad and Tobago/Barbados and in each of three study sites in Haiti). Based on guidance from the technical advisory group, 20 interviews with MSM were conducted in each site as they were expected to be more socioeconomically diverse than FSW and transgender women.

Structured interview guides covered experiences of GBV in a variety of settings and included a list of closed-ended questions about participants’ specific experiences (such as whether they had been assaulted or received negative or stigmatizing comments), as well as open-ended questions such as what types of violence they experienced, the location and perpetrators of that violence. Additional questions were asked regarding to whom they disclosed GBV, what services they sought, and what types of support they wanted to receive. Participants were asked about violence they had experienced as members of the three study populations; time frames were not specified so that participants could share experiences of their own choice regardless of when they occurred. To make participants comfortable and be responsive to the potential psychological consequences of discussing traumatic experiences, participants were given the option of not answering any questions they felt uncomfortable with, though few opted not to fully answer questions.

Development of the interview guides was informed by existing research on GBV experienced by FSWs, MSM, and transgender women, as well as the validated World Health Organization Violence Against Women and Girls instrument [38]. We developed the guides in conjunction with the study’s regional technical advisory group and guides were reviewed by and piloted with individuals from the Global Network of Sex Work Projects, the Global Forum for MSM and HIV, and the Innovative Response Globally for Transgender Women and HIV, as well as KP members in each country [39]. Following piloting, the guides were further revised to improve clarity and relevance of the questions, accuracy of the translation, and question flow.

All interviews were conducted by peer data collectors who interviewed study participants of their respective KP group (FSWs, MSM, or transgender women). Data collectors, recruited from local KP organizations, were trained in qualitative research, interviewing skills, study procedures, and research ethics, and were supervised by local researchers. Study participants were recruited by peer data collectors from KP-focused civil society organizations’ offices or during outreach activities with FSWs, MSM, and transgender women in each study country. All data were collected in 2016. All participants were 18 years of age or older and were either 1) cisgender women who reported selling sex; 2) cisgender men who reported having sex with other men; or 3) transgender women who either self-identified as transgender or who, in responding to a two-question participant eligibility questionnaire, noted that they were assigned male sex at birth and now identified as women. Individuals currently being detained by the police or awaiting trial were not eligible for participation. Members of KP groups who worked on HIV-related interventions or conducted peer outreach activities with KPs were also excluded from the study, as they were likely to be more informed and empowered than others.

The study received ethical approval from the FHI 360 Protection of Human Subjects Committee; the El Salvador National Ethics Committee on Health Research; The University of West Indies Faculties of Medical Sciences Ethics Committees at Cave Hill (Barbados) and St. Augustine (Trinidad and Tobago); and the Ministry of Public Health and Sanitation National Committee of Bioethics in Haiti. All participants provided oral informed consent prior to the interview. All study staff were trained in research ethics and study procedures to ensure the confidentiality of study participants. All interviews were conducted in a private space and were audio recorded and transcribed in English, Spanish, or Haitian Kreyol, and then translated into English as applicable. Responses to closed-ended questions were also documented by the interviewer on the interview guide. Identifying information was collected by study staff only to schedule interviews and invite participants to data interpretation events. Identifying information was not written on documents that contained any information about the study, and this information was kept separate from interview guides, transcripts, notes, and audio recordings, accessible only by study staff and will be destroyed following dissemination.

Qualitative data from Barbados, Trinidad and El Salvador were coded by a team of six researchers at FHI 360 using QSR NVivo qualitative data analysis software program [40]. The researchers developed a detailed codebook, including deductive codes generated from the data collection instruments and inductive codes emerging from the data. For each country, teams of analysts independently coded transcripts and resolved discrepancies through discussion until inter-coder agreement was achieved. After that, inter-coder agreement was assessed periodically, and the codebook revised as necessary. Overall, 20% of transcripts were coded by a team of analysts to assess agreement. Following analysis from the three other countries, qualitative results from open-ended sections of interviews conducted in Haiti were coded using a structural matrix, as these interviews were shorter and provided fewer details.

Study analysts ran code reports and reduced and organized the data into themes, including supporting quotes. Data was organized to identify the settings where violence occurred, the type of violence (emotional, physical, sexual, economic and other human rights violations), and perpetrators of violence. The data were summarized separately for FSWs, MSM, and transgender women and for each country, and then summarized across participant groups. After this initial analysis was completed, an interpretation meeting was held in each country to review the data, ensure accuracy in the interpretation, prioritize results, and discuss the dissemination plans including the optimal format in which to present the data. Meeting participants included peer data collectors, study participants, local researchers, and representatives from national key population organizations, regional NGOs, ministries of health, other governmental agencies, UNAIDS, UNDP, UWI and LINKAGES. Following individual country analysis and interpretation meetings, the analysts merged and summarized the data across countries.

Responses to closed-ended questions were entered using EpiData data entry software with double data entry for accuracy, exported to STATA, and analyzed descriptively by country and KP group to produce means and frequencies of responses to demographic questions and questions on the most common settings, perpetrators and types of GBV that participants experienced [41, 42].

## Results

A total of 278 individuals (119 FSW, 74 transgender women, 85 MSM) were interviewed across the four countries (Table 1). Participants were on average 29 years old and most frequently had attended or completed secondary education; slightly less than half of participants reported paid employment, which could include sex work.

**Table 1:** Participant demographics

	All KP groups (*n* = 278) N, mean or %	FSW (*n* = 119) n, mean, or %	TGW (*n* = 74) n, mean, or %	MSM (*n* = 85) n, mean, or %
Country^a^				
Trinidad	30	8	10	12
Barbados	20	7	5	8
Haiti	178	89	44	45
El Salvador	50	15	15	20
Age (years)^b^	29.2	30.4	29.5	27.1
Highest education level^c^				
None	4.0%	8.6%	1.4%	0.0%
Primary	20.1%	30.2%	16.4%	9.4%
Secondary	62.4%	56.0%	71.2%	63.5%
University/technical	13.5%	5.2%	11.0%	27.1%
Paid employment^c^	48.0%	57.8%	46.6%	35.7%
Ever engaged in sex work^c^	48.9%	100.0%	60.6%	38.7%

^a^ Participants per country is reported as number

^b^Age is reported as a mean

^c^ Education, paid employment and ever engaged in sex work are reported as percentages

We found some variation in participants’ educational status and employment by KP group. Fourteen percent of participants overall had attended university or technical school, while nearly one-third of MSM participants had achieved this education level (27%). FSWs were most likely to have no education, with 9% reporting this compared to 1% of transgender women and no MSM. Transgender women had the highest rate of self-reported paid employment in Trinidad and Tobago/Barbados and El Salvador (80 and 33% respectively, data not shown) while in Haiti 39% reported paid employment, considerably lower than FSWs at 67% but slightly higher than MSM at 33%. All participants in El Salvador reported markedly lower levels of paid employment (16%, data not shown) than participants in all other countries.

### Settings where GBV occurs

Study participants reported that GBV occurred in a range of settings and throughout their lives. Among MSM and transgender women in all study countries, nearly all study participants reported experiences of violence in their childhood homes. (FSWs were not asked, as requested by FSW stakeholders.) All participants who reported they engaged in sex work in Trinidad and Tobago/Barbados reported experiencing violence in brothels, bars and on the street. Violence in sex work settings was also universally reported by FSW and transgender women in El Salvador and MSM in Haiti. Among MSM in El Salvador and FSWs and transgender women in Haiti who reported engaging in sex work, reports of violence in sex work settings was also high. Violence was also very common in public places such as parks, streets and public transport among all participant groups and in all study sites. Health care centers and hospitals were reported as sites of violence by more than three-fourths of participants overall, with transgender women and MSM experiencing violence in this setting slightly more often than FSWs and participants in El Salvador reporting more violence than in other study countries. Police stations, were another commonly reported location of violence especially for transgender women; again, violence in police stations was more common in El Salvador than other study countries. Finally, violence in both schools and churches or other religious settings was reported by approximately three-quarters of MSM and transgender women. Overall, while all groups experienced violence in numerous settings, transgender women reported experiencing violence in more places than FSWs or MSM. Only five individuals (three FSW and two MSM) reported not experiencing GBV in any setting. More than three-quarters of participants reported experiencing violence in four or more settings with FSWs reporting experiencing violence in the fewest settings and transgender women the most. One of these individuals was an MSM who reported that he *“avoided”* GBV because he did not *“portray”* himself as gay in public.

### Types of violence reported

Nearly all participants reported experiencing emotional violence. Emotional violence included psychological and verbal abuse, threats of physical or sexual violence or harm, coercion, controlling behaviors, name calling and insults, intimidation, isolation and bullying. Economic violence was reported by more than three-quarters of transgender women and FSW and nearly two-thirds of MSM. This included the use of money or resources to control an individual or harm them economically, blackmail, refusing individuals the right to work or taking their earnings, (including sex work clients refusing to pay for services) and withholding resources as a punishment. Physical violence and other human rights violations were each reported by approximately three-quarters of each study population across all study sites. Physical violence included physical abuse as well as kidnapping, being forced to consume drugs or alcohol and being subjected to invasive searches. Sexual violence included: rape, coercion or intimidation to engage in sexual activity against one’s will and refusal to wear a condom. Other human rights violations included denial of basic necessities, arbitrary detention, arrest or threat of arrest and denial of health care. Notably, more transgender women reported experiencing emotional, physical, and human rights violations compared to other groups, while FSW reported economic and sexual GBV more frequently. Although nearly all MSM reported experiencing emotional GBV, the other types of GBV were reported somewhat less frequently compared to the other population groups with approximately two-thirds of MSM reporting economic, sexual, physical and human rights violations.

### Perpetrators of violence

Perpetrators of GBV included individuals that participants were closest to such as family and partners, as well as those with whom they had more limited contact. Family members, typically male, and including immediate relatives such as parents, brothers and grandparents as well as uncles and cousins, were common perpetrators of violence against participants, especially when the participants were young. Friends, peers, neighbors and community members were also commonly mentioned. Along with people they knew, participants reported that strangers, typically men encountered in public places, also perpetrated violence against them. MSM also reported that members of the LGBT community enacted violence against them. In Haiti *“vagabonds”* or charismatic and potentially dangerous men who cruise public areas perpetrated violence against both MSM and transgender women. Intimate partners, both current and former, were also commonly noted. For FSWs, the fathers of their children were mentioned. Among participants engaging in sex work, clients, other sex workers—usually those working in the same establishments—and, less commonly, people sex workers worked for such as brothel or bar owners or members of their family, perpetrated violence. Health care workers, including doctors, nurses, and staff such as receptionists were identified along with other patients, though this last group was less common. Police, and less often, soldiers and other uniformed personnel were also named; they were typically male, though women were noted in some cases. Religious leaders and members of religious communities commonly perpetrated violence, especially against transgender women. Finally, teachers were named as perpetrators of violence against MSM and transgender women during childhood and young adulthood while principals and teachers enacted emotional violence against adult FSWs when then interacted with their children’s schools.

### Consequences of experiencing GBV

When asked about the consequences of GBV, participants most commonly reported ***emotional distress*** including feeling “*sad,” “fearful,” “angry,” “hurt,” “uncomfortable,” “humiliated,” “embarrassed,” “resigned,” “overtaken,” “guilty,” “isolated,” “worthless,” “useless,” “suicidal”*, less trusting, and less self-confident. Experiences were described as *“traumatic”* and *“damaging”* and participants thought “*no, this isn’t right.”* Some participants described feeling trapped and depressed:

*“It affects me up to this day in a way that I don’t show it but, it does, because it put me into a shell and it lowered my self-esteem and […] I feel less than a woman…me personally, sometimes I doan [don’t] have no hope, there is no escape, it’s like a bond, I mean like a prison you can’t get out of.”*

- FSW, Barbados

Participants also described how their experiences negatively impacted their relationships with other people, such as their partners, colleagues, neighbors, and especially their families, including feeling as though they were not part of their family, feeling as though they were not equal to other family members, or feeling as though they were less of a person.

Fears of future GBV led to ***restricted movement and behaviors*** such as participants isolating themselves or changing their day-to-day routine to avoid certain people or places, or changing the way they walked, spoke, or dressed to avoid negative attention.

*“Eventually, eventually, you know, with verbal abuse sometimes as it becomes so constant the individual tends to place themselves within a box, right. So that, you know, they don’t venture out that box into society where they feel, you know, that their life is more in danger…I tend to prefer staying where I would be more comfortable as opposed to venturing out into public and society, where society would deem you unfit, would, they would look at you like at you as you were less than, you are not human.”*

– Transgender woman, Trinidad and Tobago

Participants, particularly sex workers, reported a range of ***economic consequences*** of GBV. Some had to leave establishments where they worked or move to another location. Some had trouble meeting their basic needs after bosses or police made them pay fines or bribes, or after a client or brothel owner withheld payments they were due. Participants who experienced GBV in childhood reported they ran away from home or were thrown out of their homes, and a few others tried to do so or were threatened with withdrawal of support. This was particularly common in El Salvador. Additionally, a small number reported having to drop out of school after their parents withdrew economic support.

*“My mom would say […] ‘If I had an effeminate son,’ she would say, ‘I would put him into the army so that they would make him a man. I would hit him, I would tie him to a tree, I would kick him out. I would never want a son like that.’ So then when I was little, I used to hear all those comments that my mom said. […] It was because of that that I had to leave home, because I felt that when they realized it, well, they were going to kick me out, and to avoid that I ran away.”*

-Transgender woman, El Salvador

FSWs in El Salvador described that when fathers of their children withheld economic support, the respondents did not have enough money to care for their children. Partners also asked participants for money or withheld money they owed to help with children. Some participants, especially transgender women, were unable to gain or maintain employment because of their gender expression.

*Interviewer: Reflect on what you just told me [is there] anything you’d like to tell me more about [when] you were applying for a job?*

*Participant: I didn’t- I don’t have the tangible evidence to prove that…*

*Interviewer: You don’t, but you always knew?*

*Participant: I always knew. I just didn’t have the substantial evidence to prove it. But I knew based upon their actions and expressions. Facial expressions and gestures. I could vouch that with them that I was trans and you would see the ‘oohs’ and the ‘ahhs’ and the facial expressions. And you knew that you wouldn’t get the job and they just didn’t call.*

–Transgender woman, Trinidad and Tobago

Some participants reported ***physical and sexual trauma*** for which they had to seek medical care, including knife and gunshot wounds, STIs, burns, miscarriage, pelvic hemorrhaging, bruises from being thrown from a car, and losing consciousness.

*“It was bad, I had to throw myself from a car, because the guy forced me in, because he thought he had taken a biological woman with him…So, when we were driving, he realized that I am a trans woman…and, yeah, he told me he was going to take me somewhere to kill me.”*

– Transgender woman, El Salvador

A few participants reported attempting suicide. One FSW from El Salvador described losing her pregnancy after being gang raped; she described this experience as her *“biggest failure.”*

Gender-based violence in health care facilities, by police, and in public institutions restricted respondents’ ***access to legal, health, and other social services.*** Respondents reported that their own and their peers’ negative experiences with providers—including encountering providers who disregard KP members’ medical or legal needs, refuse to provide them with services, make them wait longer than others, or emotionally, physically, or sexually abuse them—limited their willingness to seek services. These experiences also resulted in participants leaving services before getting care or caused them not to report crimes or made them only attend known providers that they could trust.

*“Yes. There was a time I went out with a client. We were involved in a conflict and I went to the police station to make my complaint. The officer told me if I wasn’t out so late this wouldn’t have happened, and he told me to come into the back to relay my statement, and he forced his self onto me also*.”

– FSW, Barbados

*“They told me that whether you gay or not right, umm, if you innocent, because you gay you guilty, one officer said that to me when we were arrested the first time.”*

– MSM, Trinidad and Tobago

Some participants also shared that the process of coping with GBV led to ***positive outcomes*** including increased resilience and empathy. A few FSWs and transgender women in El Salvador and Trinidad said they learned to *“depend on themselves,” “value themselves,”* and *“open a part of their identity they had been suppressing.”* Some participants reported that their experiences made them realize they should treat others with respect and avoid judging people, or that the hardships they faced made them want to help others who may be going through the same thing.

“*So far, it brings up certain hurts and pain you rather forget and leave in the past, yet still I would like if I could help somebody along the way so they could learn from my experience.”*

– MSM, Trinidad

A small number of participants reported that GBV had not had an impact on them. One MSM in El Salvador said: *“[it] makes no difference what people say [because] I accept myself the way I am.”* A few FSWs did not identify as victims and reported being empowered to stand up against discrimination.

### Disclosing GBV

Participants most often shared their experiences of GBV with a trusted friend or family member, or with another sex worker, MSM, or transgender woman. Participants felt supported when people expressed concern, empathized, shared similar experiences, encouraged and reassured them, or just listened. An FSW in Barbados said that sharing with a colleague made her feel supported because she “*actually could understand where I was coming from*”. Some participants appreciated receiving advice or instrumental support such as information about filing reports, referrals to support services, or being bailed out of jail, while some also noted that they appreciated when people respected their decisions and did not pressure them into seeking services.

Participants did not feel supported when people minimized their experiences *“She started laughing and said, ‘bad luck’”* (transgender woman, El Salvador); told them to ignore the violence, defend themselves, or avoid the setting or perpetrator; or blamed the victim for instigating the GBV. These types of negative interactions happened even when KPs disclosed experiencing GBV as children; participants reported that after disclosing sexual GBV that happened to them before the age of 18, family members did not believe them, blamed them, or even beat them.

A few participants said that they disclosed their experiences not to find support for themselves, but to support others experiencing GBV:

*“For me, to talk about certain situations, there’re people out there [….] under the LGBT or trans that would need to know that somebody has been through it [and] is there to help them […] who cares and who would understand.”*

– Transgender woman, Barbados

Many participants chose not to disclose GBV they had experienced because they felt guilty or ashamed, did not want to re-live their experiences, did not want to out themselves or reveal that they were engaged in sex work, or were afraid of punishment or further discrimination. Participants reporting GBV from a partner often described not disclosing because they felt it was a private matter or because they felt it was not significant enough to share. Sex workers said they did not disclose GBV because they were afraid of losing their job, especially when GBV came from brothel or bar owners. Some did not disclose because their perpetrators had threatened them with more violence if they told anyone. Others had accepted GBV as a part of life:

*“There comes a moment when maybe you get used to it and maybe you say that you’ve received as much as you can from life. So, many experiences that I have experienced, now they seem normal to me. So then, who is going to solve it for you? What are you going to talk about it for? What solutions are they going to give you? You know that they don’t expect it if you mention it. So then why are you going to mention it?”*

– MSM, El Salvador

When asked about disclosing GBV in a health care context, only one-third of participants reported ever being asked by a healthcare provider about GBV, and slightly less than this shared their experiences with providers. Some participants saw their GBV experiences as irrelevant to their health care; others said the GBV they experienced was personal, and they did not want to share with providers. Participants also talked about healthcare providers being untrustworthy, inattentive, insensitive, discriminatory, or unable to address their problems. Fewer transgender women and MSM described sharing their experiences with a health care worker compared to FSWs.

### Service seeking

Some participants reported seeking counseling, legal, and healthcare services for the GBV they experienced and a few reported that receiving services that were helpful including: counseling that helped them to process the GBV they experienced or medical care for physical injuries. In a few cases police or legal actions lead to perpetrators being arrested and serving jail time. A few mentioned that social norms were changing slowly and that work from key population advocacy groups and civil society organizations was helping to make progress in ensuring people were treated equally. This was mentioned most often in El Salvador.

*“Well, the positive thing is that nowadays, the NGOs provide workshops for the national police, the soldiers, the metropolitan police…[…] They are starting to take the LGBTI community into account more. […] You can see that they’re talking about us on the news. […] Nowadays it’s spreading, we're not as, you know, singled out. We're a bit more visible nowadays, we're taken into account more. You could say that things are progressing”*

– MSM, El Salvador

Unfortunately, however, the majority of participants who talked about seeking services said that services did not meet their needs, or they were further victimized by service providers. Healthcare staff told an FSW in El Salvador *“that it [an experience of GBV] happened to me because I am a street whore, and that if I were a respectable woman then that wouldn’t have happened.”* The police told another FSW after she was raped: *“that’s what you get for working in the street.”* People who reported GBV to the police usually said no legal action took place as a result.

Most participants did not seek any services for the GBV they experienced. Participants said they did not think they needed services because their experiences were not severe enough, they did not think they would get the help they needed, getting help was too burdensome, or they did not know services were available. A transgender woman from Barbados said she did not *“feel like there’s anything the police could really do.”* Participants were also fearful of being outed and experiencing discrimination from service providers:

*“[When] people do something to me, I don’t go to the police. Because I already see that both the police officer and the judges that are working in the public institutions, they humiliate people like me a lot.”*

– Transgender woman, Haiti

*“They already assume that you’re guilty and that you were the one who initiated everything, the culprit, the criminal. Never the other person. It unconsciously makes you feel like you’re guilty. I was scared. I said, ‘I don’t want to report it, I don’t want to be asked if I’m homosexual.’”*

– MSM, El Salvador

Participants identified unique challenges to accessing services after experiencing GBV when they were under the age of 18. They said they were too young to seek services by themselves, could not travel to services by themselves, or were too young to understand that they needed help. An FSW from El Salvador said, *“at the time, I knew nothing”* and wasn’t aware she could report the abuse; another FSW from Barbados said that she did not seek services because *“I dismissed it [the experience of GBV] mentally”* while another FSW in Barbados said she was going to seek services, but then decided it was “*too much of work*.” Participants who did receive services for sexual GBV experiences under the age of 18 reported their families were instrumental in responding to and seeking services.

### Services wanted

Despite limited or unhelpful service-seeking experiences, participants expressed a desire for additional GBV services, most commonly mental health services like counseling or support groups. Many participants, especially in Haiti, also stated the need for healthcare services more generally. Some wanted better police services, and participants from El Salvador and Haiti specifically mentioned employment opportunities or assistance getting jobs as important for preventing or recovering from GBV. Participants emphasized that services should be KP-friendly and safe, and service providers should be respectful, supportive, accepting, and protect clients’ privacy and confidentiality:

*“I would like for the police to pay more attention to you and to help you the way they should, just like with any other person, treat you the same. That they should help you like they are supposed to. Same goes for health, that they should help you, not discriminate against you, not single you out for who you are. They should treat you like a regular person, normal, just like everyone else who is waiting there at the clinic.”*

– Transgender woman, El Salvador

*“I would like it to be taught at the police academy that they should respect people’s rights, that they should know everyone is a person and everyone is free, they have their own choices. They should be taught to respect people’s rights.”*

– Transgender woman, Haiti

More than half of participants said they would like healthcare workers to ask clients about GBV so that providers would better understand clients’ needs and provide better quality care, including mental health services, referrals to GBV services, and access to safe spaces. There was variation among KP groups, however, with slightly less than three-quarters of FSWs in all countries reporting a desire for healthcare workers to ask, with half of transgender women reporting they wanted health care workers to ask and less than half of MSM reporting the same. There was also variation between countries from more than three-quarters of FSW in El Salvador wanting providers to ask, compared to a low of approximately one-quarter of MSM in Haiti. Some said that asking about GBV was part of healthcare providers’ job and would show that they care about their clients but stipulated that providers should keep clients’ information confidential. Participants in El Salvador explicitly stated that providers asking clients about GBV could reduce perpetration of GBV within the health care system, change attitudes towards stigmatized groups, and encourage key populations to seek care.

### Perceptions of HIV risk

Across study settings and participant groups, less than one quarter of participants thought GBV increases risk of HIV infection; this was much lower in Haiti compared to other study countries. In Trinidad and Tobago, Barbados and Haiti transgender women were the most likely to identify GBV as a risk for HIV while in El Salvador, FSWs were most likely to see the connection between GBV and HIV infection. Many respondents reported that their HIV risk came from personal choices, such as not wanting to use condoms, or accidents, such as broken condoms, but did not link their risk to GBV. Some explained that they did not think GBV increased their HIV risk because they always used condoms or because the GBV they had faced was not sexual in nature. Participants who believed that GBV increased their HIV risk reported healthcare providers do not help KP members or are otherwise violent toward them, limited service seeking, and experiences of sexual GBV could result in HIV infection. One transgender woman noted that the feelings of isolation she had because of discrimination made her more likely to agree to unprotected sex in order to feel companionship. FSWs, more so than transgender women and MSM, linked GBV with increased risk of HIV because of clients or other perpetrators who refused to wear condoms. This was brought up most frequently in El Salvador:

*“When you engage in sex work, you’re really exposed to all types of diseases, even more so when you are forced to have sexual relationships without protection, that’s really a factor that could result in you being infected with HIV.”*

– Transgender woman, El Salvador

### Limitations

While data from all countries were included in the analysis, the data from Haiti contained much less information than in other countries. We hypothesize this could be due to increased levels of stigma around issues of gender identity and sexual orientation leading to a reluctance to openly discuss these issues. Additionally, peer data collectors in Haiti were less experienced with research and did not probe participants for more detailed answers as much as in other countries. Furthermore, as transcripts were translated from Haitian Kreyol to English, and some of the translations were unclear. Participants’ identification as transgender varied by country; while there was a strong local identity of transgender women in Trinidad and Tobago, Barbados and El Salvador, the presence of a transgender identity was relatively limited in Haiti and transgender women often referred to themselves as MSM. We worked with the local research team in Haiti to develop terminology and ways of asking about gender identity that spoke to the local concept of transgender women, though these participants rarely openly identified as women.

This study did not specifically explore how GBV affects HIV-positive KP’s ability to access care and adhere to HIV treatment, important considerations for improving KP’s health beyond the scope of this research. Finally, the findings offer insight to common experiences of GBV faced by these populations, but given the purposive sampling and qualitative approach, the results are not generalizable to broader population groups’ experiences either within countries or across the region.

## Discussion

Our study found that FSW, MSM and transgender women faced GBV throughout their lives in a wide range of settings. GBV was largely perpetrated by those who were meant to support and protect KPs, including family members, health care workers, and police as well as the wider community and strangers throughout their lives. Violence took many forms and included not only sexual and physical violence but emotional, economic and human rights violations as well. We also found that KP members’ perceptions of violence, current responses to the violence, and desires for violence prevention and response provide a foundation on which HIV programs can integrate GBV and HIV services. These findings expand the breadth of the current literature by describing the types, settings and perpetrators of violence across the life course [34, 43, 44].

For MSM and transgender women, violence starts in childhood and, for all participants, violence extends throughout their public and private lives. Transgender women faced particularly high levels of violence. Gender-based violence was so pervasive that many KPs perceived it as a regular part of their daily lives and not a violation of their human rights. If they did recognize GBV as unjust or illegal, many felt powerless to stop it. Participants recognized the negative impact of GBV on their mental and physical health as well as their relationships, economic stability, and ability to move freely but generally not on their HIV risk other than in the case of rape or unprotected sex. Many expressed desire for services and support to help cope with and prevent GBV, including being asked about violence by health care providers and a desire for services to cope with and prevent GBV.

Though many participants disclosed experiences of GBV, they rarely sought services from health care workers or police following violence. Those who disclosed GBV were often met with blame, advice to restrict their own movement, or the observation that there was nothing to be done. At the same time, KP members recognized and articulated the types of support they wish to receive upon disclosure: empathy, information on services, and equal treatment.

While study participants generally did not identify a link between GBV and HIV outside of sexual violence, the consequences of GBV that participants described have clear implications for KP members’ ability to receive information or services that could help them prevent, detect, and treat HIV and other sexually transmitted infections. These consequences include relationships with healthcare providers and police marked by discrimination, stigma, and concerns over confidentiality; a limited ability to report GBV and receive services from police or lawyers; lack of access to appropriate and acceptable health care; limited or constrained economic lives; disempowerment and hopelessness that limit a desire to seek care; and isolation and restricted freedom of movement that can impede physically reaching services.

This research makes several key contributions to the literature. Using a peer-lead approach coupled with substantial engagement from KPs allowed for a through discussion of sensitive topics with hard-to-reach populations including the opportunity for KPs to both share their experiences of violence and their perspective on the services they would like to address and prevent violence. We identified a range of settings where violence occurs, perpetrators and types of violence faced by KPs across their lifespan. Previous quantitative research from sub-Saharan Africa has documented GBV perpetrated by different groups including police, partners, family, and community members [45–52]; but qualitative research to contextualize these experiences and provide data specific to the LAC region is currently lacking [28, 53, 54]. Similarly, few studies to date have taken the approach of documenting experiences of violence across the life course among populations at risk for HIV [28, 55, 56] or documenting early experiences of GBV among KP groups [54]. Finally, this study provides information on the gender-based origins of violence which will enable KP programs to build on existing initiatives to address GBV in the general population and to more systematically integrate gender into KP programming.

This study’s results reinforce previous research which note that gender-based discrimination including discrimination based on sexual orientation, gender expression and gender non-conformity, results in violence [43, 57–59]. Study participants report multiple examples of violence triggered by violation of gender norms and these experiences point to the need to address the deep-rooted gender norms behind GBV. Recognizing gender-based origins of violence also opens opportunities to collaborate with organizations addressing GBV in the general population [60].

The link identified between experience of GBV and subsequent service seeking suggests that HIV programs should integrate GBV prevention and response to improve their effectiveness. And, indeed, global and regional strategy and guideline documents state that violence should be addressed as part of the HIV response for KPs [61, 62], but these policies must be translated into concrete practices to prevent and address violence at the global, regional, and national levels. Informed by this study, the LINKAGES project has developed guidelines for integrating services to address violence with HIV prevention and treatment. This guidance details how violence should be prevented, detected and responded to as part of outreach and clinical services for KPs; it also describes work with civil society and law enforcement to create an enabling environment in which KP members understand their rights and can seek support from the police (Personal communication, Robyn Dayton, Technical Advisor, LINKAGES). In line with the study findings, the guidance details how gender-based origins of violence should be directly addressed, a departure from some KP programs that have often failed to examine issues affecting key populations using a gender lens [63].

Working with health care providers and police in the context of an HIV program is logical as these actors are both service providers and perpetrators, are more accessible than family, intimate partners, or even clients and among the most common perpetrators of violence. These groups are also well-placed to help individual KPs begin to see violence as a rights violation and violence must be treated as a violation and not the fault of the victim to reduce the likelihood of revictimization upon disclosure. As the WHO notes in their 2003 guidance on caring for those who have experienced violence, *“Many survivors of sexual assault have described the kindness of the treating personnel as being beneficial to their recovery. Conversely, many describe comments made by police, doctors, counsellors and other persons with whom they have had contact as a result of the assault that have haunted them for years”* [64]. The LINKAGES project conducts gender transformative trainings with police, health care workers and peers to sensitize these groups to the needs of KPs and provide them with skills in first-line support tailored to key populations [65].

Providing documentation of the violence faced by KPs is also essential. At the regional level UNDP, has worked with regional and local civil society organizations to support KPs to document violence and record human rights violations and implement a monitoring system to provide data on violence and demand attention from governments. Finally, government partners, civil society organizations, UNAIDS and UNDP have developed “zero discrimination” indicators for countries in Latin America and the Caribbean to report regionally on violence and HIV among transgender women [66, 67].

Responses to the findings of this study will require political will. The frequency and pervasiveness of GBV and lack of service seeking described in this study indicate a clear need for government and civil society to do more to protect vulnerable populations from human rights violations. Data from this study provide concrete country- and population-specific data on violence faced by KPs that will help programmers working with violence, community-based organizations and advocates to highlight and address human rights violations faced by KPs.

## Conclusion

In Latin America and the Caribbean, where levels of GBV in some countries are high among the general population, decision-makers and others may not be aware that those who are the most marginalized—including FSWs, MSM, and transgender women—require specific interventions and support. Without addressing the GBV that members of KPs experience, an effective response to HIV will remain out of reach. Our study found that across the four study countries—varying widely in cultural, social, and legal systems—KPs experienced GBV from state and non-state actors, received limited support, and experienced a wide range of impacts that affected HIV service uptake. Through coordinated interventions that address both HIV and GBV against KPs, this region has the opportunity to improve both KPs’ overall well-being and the national burden of HIV while respecting each individual’s humanity and helping each reach her or his fullest potential.

*“…My final comment would be that above all, we need to be recognized as human beings. We are women that pay the Municipal Council taxes, we pay for our homes, we pay for our telephone, we pay for our water, we pay for our electricity, we pay taxes, even for a pound of salt. And I think that the same taxes I pay, a Municipal Council employee or a cafeteria worker or a civil servant pays the same. I think that we are all equal. I don’t feel that I am better or worse than any other person.”*

– FSW, El Salvador

## Abbreviations

FSW: Female sex worker
GBV: Gender-based violence
KP: Key population
LINKAGES: Linkages across the Continuum of HIV Services for Key Populations Affected by HIV Project
MSM: Men who have sex with men
UN: United Nations
UNAIDS: Joint United Nations Programme on HIV/AIDS
UNDP: United Nations Development Programme
USAID: United States Agency for International Development
UWI: University of the West Indies
